# Long-term survival of femoral neck fracture patients aged over ninety years: Arthroplasty compared with nonoperative treatment

**DOI:** 10.1186/s12891-020-03249-7

**Published:** 2020-04-08

**Authors:** Yang Liu, Chong-wei Zhang, Xiao-dan Zhao

**Affiliations:** 1 Department of Orthopedic Surgery, National Clinical Research Center for Geriatrics, West China Hospital of Sichuan University, Wai Nan Guo Xue Lane No. 37, Wuhou District, Chengdu, Sichuan Province P.R. China 610041; 2 Department of Laboratory Medicine, West China Hospital of Sichuan University, Wai Nan Guo Xue Lane No. 37, Wuhou District, Chengdu, Sichuan Province P.R. China 610041

## Abstract

**Background:**

The aging of the Chinese population is expected to lead to an increase in nonagenarians and centenarians. The mortality rate in nonagenarian hip fracture patients is equivalent to the mortality rate in the average population at 5 years after injury. It is imperative to evaluate 5-year mortality in this small but very challenging subgroup of patients to optimize patient management. The primary purpose of the current retrospective study was to compare five-year survival in patients aged over 90 years who received arthroplasty or nonoperative treatment for femoral neck fracture during a 16-year period.

**Methods:**

From January 1998 to December 2014, all consecutive nonagenarian and centenarian patients with femoral neck fracture admitted to our hospital were included in the evaluation. The primary outcome was defined as thirty-day, 1-year, 3-year, and 5-year mortality after injury. Survival analysis was performed with the Kaplan-Meier method. Using the log-rank test, stratified analyses were performed to compare differences in the overall cumulative mortality and mortality at three time points (1 year, 3 years, and 5 years) after injury and differences in survival distributions.

**Results:**

Over the 16-year study period, the arthroplasty group and the nonoperative treatment group included 33 and 53 patients, respectively. The long-term survival probability of the arthroplasty group was significantly higher than that of the nonoperative treatment group (*p* = 0.002). The survival time of the arthroplasty group was significantly higher than that of the nonoperative treatment group (median (P_75_-P_25_) = 53 (59) versus median (P_75_-P_25_) = 22 (52), *p* = 0.001). The mortality differences, except for 30-day mortality, at five time points (1, 2, 3, 4, and 5 years) between the nonoperative group and arthroplasty group were significant. The stratified analyses of overall cumulative mortality and mortality at three time points (1, 3, and 5 years) after injury demonstrated that the nonoperative treatment group had significantly higher cumulative mortality than the arthroplasty group.

**Conclusions:**

Our study demonstrates that arthroplasty is more likely to improve long-term survival in femoral neck fracture patients aged over 90 years than nonoperative treatment. It can be expected that nearly half of patients will survive more than 5 years after surgery.

## Background

The aging of the Chinese population is expected to lead to an increase in nonagenarians and centenarians. Chinese people comprised approximately 15% of the global population over 90 years of age at the end of 2017 [1]. Mortality in people aged over 80 years reaches a “plateau” after age 105 [2]. Thus, the extended life expectancy of elderly individuals profoundly influences many aspects of medical care.

Hip fracture ranks among the top 10 causes of disability and mortality in the elderly population [3]. Intracapsular femoral neck fractures account for 50% of all hip fractures [3]. The age-specific incidence of hip fracture in elderly Chinese individuals has increased during the last decade [4, 5]. Accordingly, femoral neck fracture in extremely elderly individuals in China represents a tremendous burden on the public health system.

Although extensive studies on 30-day or 1-year mortality in femoral neck fracture patients aged above 90 years exist, very little data on 2-year or 5-year survival in these patients exist [6–9]. The additional life expectancy for nonagenarian hip fracture patients is 4–5 years [10, 11]. Moreover, recent studies demonstrated that the mortality rates in nonagenarian hip fracture patients returned to a rate equivalent to that in the average population at 5 years after injury [9, 12–15]. Therefore, it is imperative to evaluate 5-year mortality in this small but very challenging subgroup of patients to optimize patient management.

Currently, there is no consensus regarding the long-term survival of hip fracture patients over 90 years of age. Few articles have discussed five-year survival in nonagenarians [9, 16, 17]. Furthermore, less than 5% of patients survive for more than 10 years [16]. Thus, several authors considered 5-year survival as a long-term survival result in this extremely elderly population [17].

The primary purpose of the current retrospective study was to compare five-year survival in patients aged over 90 years who receive arthroplasty or nonoperative treatment for femoral neck fracture during the same 16-year period.

## Methods

From January 1998 to December 2014, all consecutive nonagenarian and centenarian patients with femoral neck fracture admitted to our hospital were included for evaluation. Patients with polytrauma, open fractures, pathological fractures, femoral head fractures, subtrochanteric fractures, or intertrochanteric fractures were excluded from this study. The follow-up endpoint was defined as the date of death or emigration or 1 April 2019, whichever came first, and survival was determined at this time. The institutional review board of our academic hospital approved this study.

The goal of care discussion was typically attended by the patient, their family, and representatives from the medical hip fracture co-management service and orthopedic surgery unit. The purpose of this discussion was to listen to the patient to understand their previous and current quality of life and ensure that an informed decision was being made. Nonoperative management was undertaken only when requested by the patient or their family.

According to the different treatment regimens (nonoperative treatment versus arthroplasty), we divided the patients into the nonoperative group and arthroplasty group. Seven senior surgeons performed the arthroplasty procedures (including total hip arthroplasty and hemiarthroplasty).

### Covariates

The collected data included age, sex, injury side, comorbidities, length of stay, body mass index (BMI), in-hospital complications, transfusion amount, hyperthermia, intensive care unit (ICU) stay, mortality date, and 30-day readmission obtained from the hospital’s patient management system. Detailed definitions of admission laboratory parameters are available in the User Guide for the ACS NSQIP Participant Use File [18].

### Comorbidities

Comorbidities on admission were assessed by the Charlson comorbidity index (CCI) [19] and the American Society of Anesthesiologists (ASA) physical status classification [20]. The CCI determines the comorbidity level according to the number and severity of 19 predefined conditions. The CCI is the most popular tool for evaluating comorbidities in clinical studies. Because the study participants included only patients aged over 90 years, the age-adjusted variant of the CCI was not used. The ASA grading scale includes five classes, from Class I to Class V. No patient in this study was graded as Class V. The ratings were divided into two categories: Class I or II and Class III or IV.

### Complications

Complications were categorized as cardiac, pulmonary, gastrointestinal, urologic, or cerebrovascular. Cardiac complications included acute myocardial infarction, arrhythmia, congestive heart failure exacerbation, and unexplained hypotension. Pulmonary complications included acute respiratory failure, prolonged intubation, and pneumonia. Gastrointestinal complications included obstruction, perforation, and bleeding. Urological complications included urinary tract infection and urinary retention. Cerebrovascular manifestations included cerebral vascular accident and pulmonary embolism. Each type of complication was then transformed into a bivariate variable to define whether a patient did or did not have the complication.

### Mortality data

The mortality date of the participants in both groups was obtained from the National Public Security System (NPSS) and the three primary health insurance schemes in China: Urban Resident Basic Medical Insurance (URBMI), Urban Employee Basic Medical Insurance (UEBMI), and the New Rural Cooperative Medical Scheme (NRCMS). These register systems include the survival status of all citizens and are updated annually. If the mortality date was not retrievable from the system, it was retrieved through local community registration charts, nursing homes, or telephone follow-ups. We defined short-term mortality as death occurring more than 30 days but less than 12 months after femoral neck fracture and long-term mortality as death occurring 5 years after fracture. The primary outcome was defined as 30-day mortality and 1-year, 2-year, 3-year, 4-year, and 5-year mortality after injury.

### Statistical analysis

Data were analyzed with the 22.0 IBM SPSS statistical package (SPSS Inc., USA) for Windows. The results are expressed as means ± standard deviations (SDs), medians [25–75 quartiles] for nonnormally distributed quantitative data or numbers (percentages), and 95% confidence intervals. The normally distributed variables were assessed using an independent t-test and a chi-square test. When comparing two independent samples with a non-normal distribution, the Wilcoxon rank-sum test was used. Survival status between the two groups was analyzed by the Kaplan-Meier method for all-cause mortality. Using the log-rank (Mantel-Cox) test, stratified analyses of overall cumulative mortality and mortality at three-time points (1 year, 3 years, and 5 years) after injury were performed to compare the differences in survival distributions. A two-sided significance test was performed for all tests, and a *p*-value < 0.05 was considered statistically significant.

## Results

Over the 16-year study period, 89 femoral neck fracture patients aged over 90 years were admitted to our hospital. Among them, one patient was excluded because he underwent internal fixation (2.8%, 1/35,1 hip). In the arthroplasty group, one patient (2.8%, 1/35, 1 hip) was excluded from this review because they underwent arthroplasty in another hospital. The survival data of 1 patient in the nonoperative treatmentgroup (1.8%, 1/54, one hip) were not retrievable; therefore, these three patients were excluded from the final statistical analysis.

Finally, 86 patients, including 43 women (50%), and 43 men (50%), were included in the study. The arthroplasty group and the nonoperative treatment group included 33 and 53 patients, respectively. The median age of the arthroplasty group and the nonoperative group was 92 (90–104) years and 91.5 years (90–103), respectively. The baseline characteristics of the two groups are shown in Table 1.

**Table 1:** Chi-square test compares the baseline variable of the two groups with a normal distribution

Variable	Treatment regime groups		*χ2*	*P*
	Nonoperative group n(%)	Arthroplasty group n(%)		
**Gender**				
Female	27 (62.8)	16 (37.2)	0.049	0.825
Male	26 (60.5)	17 (39.5)		
**Fracture side**				
Left	28 (65.1)	15 (34.9)	0.443	0.506
Right	25 (58.1)	18 (41.9)		
**Complications**				
Pulmonary complications	32 (72.7)	12 (27.3)	3.101	0.078
Peptic ulcer disease	5 (62.5)	3 (37.5)	0.003^a^	0.958^b^
UTI	4 (57.1)	3 (42.9)	0.065^a^	0.799^b^
Cardiac Complications	6 (60.0)	4 (40.0)	0.013^a^	0.910^b^
Cerebrovascular complications	8 (100.0)	0 (0.0)	3.849^a^	0.050^b^
DVT	9 (90.0)	1 (10.0)	2.614^a^	0.106^b^
Delirium	26 (74.3)	9 (25.7)	3.999	0.046*
Pressure ulcers	20 (83.3)	4 (16.7)	6.632	0.010*
Hyperthermia	13 (48.1)	14 (51.9)	3.024	0.082
**Commodity**				
Diabetes mellitus	5 (41.7)	7 (58.3)	1.471^a^	0.225^b^
COPD	34 (69.4)	15 (30.6)	2.900	0.089
Renal disease	9 (81.8)	2 (18.2)	1.306^a^	0.257^b^
Coronary artery disease	5 (50.0)	5 (50.0)	0.210^a^	0.647^b^
Commobidity Hypertension	21 (56.8)	16 (43.2)	0.652	0.420
Commobidity UTI	2 (50.0)	2 (50.0)	0.240^a^	0.624^b^
Commobidity Stoke	9 (81.8)	2 (18.2)	1.306^a^	0.253^b^
**Admission laboratory parameters**				
Low HGB(< 12 g/dL)	30 (61.2)	19 (38.8)	0.008	0.929
Low HCT (< 30%)	14 (87.5)	2 (12.5)	5.564	0.018*
Low WBC count (< 4500/mcL)	2 (50.0)	2 (50.0)	0.240^a^	0.624^b^
High WBC count(> 10,000/mcL)	14 (66.7)	7 (33.3)	0.298	0.585
Low platelets (< 150,000/mcL)	23 (62.2)	14 (37.8)	0.008	0.929
High INR (> 1.1)	23 (71.9)	9 (28.1)	2.263	0.133
High BUN (> 30 mg/dL)	15 (71.4)	6 (28.6)	1.129	0.288
Creatinine (> 1.3 mg/dL)	9 (60.0)	6 (40.0)	0.020	0.887
Low ALB<34G/L	29 (80.6)	7 (19.4)	9.381	0.002 *
High Bilirubin (> 1.9 mg/dL)	3 (60.0)	2 (40.0)	0.006^a^	0.939^b^
High sodium (> 145 mEq/L)	1 (25.0)	3 (8.1)	1.033^a^	0.310^b^
Low sodium (< 135 mEq/L)	15 (65.2)	8 (34.8)	0.171	0.679
**Transfusion**				
Number of Transfusion Red cell	8 (30.8)	18 (69.2)	15.001	< 0.001*
Number of Transfusion Plasma	4 (30.8)	9 (69.2)	4.726a	0.030^b^*
**ICU stay**	5 (55.6)	4 (44.4)	0.001a	0.973^b^
**30-day re-admission**	8 (80.0)	2 (20.0)	0.856a	0.355^b^

* *P* < 0.05 was considered statistically significant. ***a***. The chi-square test of continuous correction was used, and the expected count of the cell was less than 5 and greater than 1. ***b***. Continuous correction chi of square test significance (2-sided)

The differences in the rates of delirium, pressure ulcers, hematocrit (HCT) < 0.30, and low albumin (ALB), as well as the number of transfusions of red cells and plasma between the nonoperative group and arthroplasty group, was statistically significant (Table 1). Other baseline variables between the two groups were not significantly different.

Subsequent stratified analyses indicated that in terms of the ASA grade, CCI score, and age, the differences between the two groups were not significant (Table 2). However, the long-term survival in the arthroplasty group was significantly higher than that in the nonoperative treatment group (39.3%,13/33 versus 11.3%,6/53, *p* = 0.002). Expectedly, the length of stay in the arthroplasty group was higher than that in the nonoperative treatment group (Table 3).

**Table 2:** The stratified analyses of ASA, CCI, age, and survival patient number of two groups

Variable	Treatment regime groups		*χ*2	*P*
	Nonoperative group n(%)	Arthroplasty group n(%)		
**ASA**				
1or2	27 (60.0)	18 (40.0)	0.056	0.813
3or4	26 (63.4)	15 (36.6)		
**CCI**				
0or1	18 (50.0)	18 (50.0)	4.508	0.105
2	11 (61.1)	7 (38.9)		
≥ 3	24 (75.0)	8 (25.0)		
**Age-stratified**				
90—94 years	41 (62.1)	25 (37.9)	0.029	0.864
≥ 95 years	12 (60.0)	8 (40.0)		
**Long-term survival probability**				
Survival > 5 years	6 (31.6)	13 (68.4)	9.31201	0.002*
Survival< 5 years	47 (70.1)	20 (29.9)		

* *P* < 0.05 was considered statistically significant

**Table 3:** The Wilcoxon Rank - Sum test for two variables without a normal distribution between two groups

Variables	Treatment regime		***Wilcoxon W***	***P***
	Nonoperative group ***Median(P***_***75***_***-P***_***25***_***)***	Arthroplasty group ***Median(P***_***75***_***-P***_***25***_***)***		
**Age**	91.5 (4.0)	92.0 (4.0)	805.0	0.530
**Length of stay**	8 (17)	18 (59)	458.5	<0.001*
**Delay of Admission**	3 (9.60)	1 (3.71)	630.5	0.053*
**Survival time (months)**	22 (52)	53 (59)	514.0	0.001*
**BMI**	17.7 (3.2)	19.1 (3.0)	582.0	0.009*
**Laboratory parameters**				
WBC	7.23 (3.83)	7.95 (3.68)	846.0	0.914
Platelets	166 (98)	161 (119)	794.0	0.475
BUN	7.51 (5.86)	6.92 (3.47)	791.5	0.461
INR	1.09 (0.11)	1.08 (0.12)	742.0	0.239
Creainine	75.50 (46)	79.60 (37)	849.0	0.821
Sodium	138.15 (7.20)	139.20 (6.90)	874.0	0.996
Billirubin	13.05 (10.60)	14.60 (7.40)	746.0	0.254

*P < 0.05 was considered statistically significant

The survival time in the arthroplasty group was significantly longer than that in the nonoperative treatment group (median (P_75_-P_25_) = 53 (59) versus median (P_75_-P_25_) = 22 (52), *p* = 0.001). Through the last follow-up, the longest survival times in the two groups was 175 months in the arthroplasty group and 93 months in the nonoperative treatment group. The Wilcoxon rank-sum test also demonstrated that the differences in BMI and serum sodium levels at admission between the two groups were significant (Table 3).

The cumulative mortality after injury in the 30-day, 1-year, 2-year, 3-year, 4-year and 5-year nonoperative group versus arthroplasty groups were 17.0% versus 9.1, 43.4% versus 12.1, 50.9% versus 24.2, 64.2% versus 39.4, 71.7% versus 45.5, and 79.2% versus 51.5%, respectively (Table 4). The overall cumulative mortality in the nonoperative group was 1.5-fold higher than that in the arthroplasty group. The difference between the two groups was significant (*p* = 0.007). The differences in mortality, except for 30-day mortality, at five-time points (1 year, 2 years, 3 years, 4 years, and 5 years) between the nonoperative treatment group and arthroplasty group were significant.

**Table 4:** The compare of cumulative mortality after injury between two groups

Cumulative mortality after injury	Treatment regime		***χ2***	***P***
	Nonoperative group N (%)	Arthroplasty group N(%)		
0-30 days	9 (17.0)	3 (9.1)	1.055	*0.304*
0–1 year	23 (43.4)	4 (12.1)	9.236	0.002*
0–2 year	27 (50.9)	8 (24.2)	6.008	0.014*
0–3 year	34 (64.2)	13 (39.4)	5.029	0.025*
0–4 year	38 (71.7)	15 (45.5)	5.923	0.015*
0–5 year	42 (79.2)	17 (51.5)	7.261	0.007*
Total	42 (79.2)	17 (51.5)	7.261	0.007*

* *P* < 0.05 was considered statistically significant

**Fig. 1:**
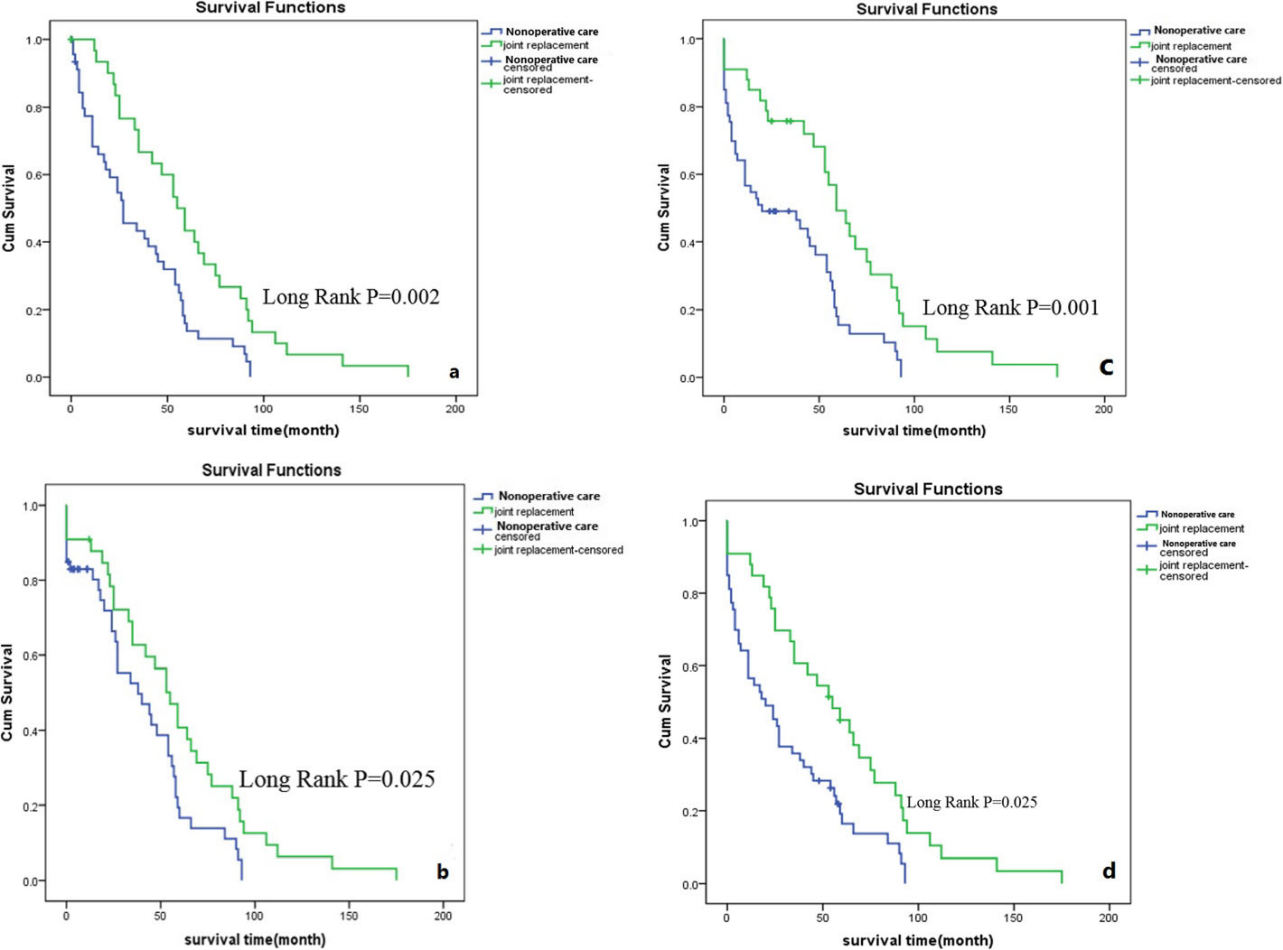
**a**. Shown the overall survival of the arthroplasty group is significant higher than the nonoperative treatment group (Log-rank P = 0.002). **b**-**d**. The 1-year (**b**), 3-year (**c**) and 5-year survival (**d**) distribution difference between two groups is significance (Log-rank *P* = 0.025), (Log-rank *P* = 0.001) and (Log-rank *P* = 0.025), respectively

Table 5 demonstrates that the survival rates in the arthroplasty group at six different time intervals (30 days, 1 year, 2 years, 3 years, 4 years, and 5 years) were significantly higher than those in the nonoperative group. The overall survival time in the arthroplasty group was also higher than that in the nonoperative group (*p* = 0.002). As a secondary analysis, survival status analysis of the two groups was performed with a Kaplan-Meier survival curve for all-cause mortality. As presented in Fig. 1, using the log-rank (Mantel-Cox) test, the stratified analyses of overall cumulative mortality and mortality at three time points (1 year, 3 years, and 5 years) after injury demonstrated that the nonoperative treatment group had significantly higher cumulative mortality than the arthroplasty group.

**Table 5:** The log-rank test for survival length during the different time interval between two groups

Different Time intervals	Treatment regime		*χ2*	*P*
	Nonoperative group ($\overline{x}\pm {s}_d$)	Arthroplasty group ($\overline{x}\pm {s}_d$)		
(0, 30]days	34.78 ± 4.26	62.00 ± 7.04	10.017	0.002*
(0, 1]year	40.01 ± 4.45	57.93 ± 7.18	5.021	0.025*
(1, 2] year	31.56 ± 4.22	62.62 ± 7.45	12.373	< 0.001*
(2, 3] year	33.56 ± 4.49	64.00 ± 7.75	11.100	0.001*
(3, 4] year	30.85 ± 4.30	58.82 ± 7.48	10.048	0.002*
(4, 5] year	30.58 ± 4.35	58.93 ± 7.80	5.021	0.025*
Total	31.36 ± 4.22	73.26 ± 11.65	9.568	0.002*

* *P* < 0.05 was considered statistically significant

## Discussion

According to our study, the cumulative five-year mortality rates in the arthroplasty group and nonoperative treatment group were 51.5 and 79.2%, respectively. Femoral neck fracture patients over 9 years of age can benefit from arthroplasty surgery. Our study suggested that patients who received nonoperative treatment had higher rates of short- and long-term mortality than those who underwent surgery. They also had a shorter mean survival times than arthroplasty patients. Additionally, the annual mortality in the arthroplasty group was significantly lower than that in the nonoperative treatment group. To our knowledge, the current study is the first in the world to assess this critical issue in a low- or middle-income country, with a timeline spanning more than 16 years.

The chance of surviving to the age of 90 has increased markedly over the last 50 years [21]. The life expectancy for 90-year-olds (ranging from 4 to 5 years) in most high-income countries is still reasonably shorter than that for 80-year-olds (ranging from 8 to 10 years) [21]. Recently, two successive cohort studies from China presented results consistent with the results from developed countries [22, 23]. Liu et al. demonstrated that the life expectancy of the population aged 90–99 years was 3.9–4.0 years [22].

Some studies have shown that the overtreatment of extremely elderly individuals is still a general concern, and potential side effects of treatment have to be balanced with the overall survival prognosis, which is generally based on the overall chance of survival to a given age or overall life expectancy [21, 24]. It has been argued that clinicians should discuss overall survival prognosis with patients aged 85 and older [24].

On the other hand, there is a lack of consensus on the definition of long-term survival for hip fracture patients aged 90 and older. Some recent studies demonstrated that the mortality rates in nonagenarian hip fracture patients were equivalent to the mortality rates in the average population at 5 years after injury [9, 12–16]. Less than 5% of patients survive for more than 10 years [16]. Consequently, the five-year survival rate can be considered a reliable parameter to reflect the long-term outcomes of extremely elderly patients with hip fracture.

During the past several decades, the dilemma of how to optimize care for extremely elderly hip fracture patients has included two aspects. First, previous reports on hip fracture patients aged older than 90 years mainly focused on 30-day and 1-year mortality and paid little attention to the survival status at 2 years or more. Second, many studies previously evaluated long-term mortality in hip fracture patients, but only a few assessed the survival of nonagenarian hip fracture patients [9, 11, 17, 25, 26].

Compared to other investigations on short-term mortality after surgery for hip fracture, our 30-day rates were similar (9.1% versus a range from 5.6 to 9.6%) [6, 10, 11, 17, 27, 28]. An exception is a Taiwan investigation in which 95.4% of patients survived to 30 days, which is the lowest mortality rate in nonagenarian femoral neck fracture patients in the literature [16]. Some studies have revealed results that conflict with those in the current study, in which 30-day mortality was higher than 10% [7, 26, 29]. These discrepancies may be partially due to different social and cultural aspects, policies, and health system services. Advances in medications, lifestyles, and socioeconomic status might also contribute to these mixed survival outcomes.

Based on national data from the Chinese Longitudinal Healthy Longevity Study, 7234 individuals who survive to 90 years of age have an all-cause annual mortality rate of 22.4–23.4% [23]. An earlier published study found that the expected 1-year survival rate for the average Japanese population aged 90 years and older was 83.7% in women and 77.6% in men [30]. In our study, the 1-year mortality rates in the arthroplasty and nonoperative groups were 12.1 and 43.4%, respectively. One of the possible reasons for this difference is the surgical treatment of the femoral neck fracture, no longer increasing the mortality compared with the general population during the last two decades [31].

Our 1-year mortality result is also consistent with another study performed in North China. In this multicenter retrospective study of 327 nonagenarian individuals, Liu et al. showed that the 1-year mortality after surgery was 11.6% [32]. Previous studies demonstrated that 1-year mortality in nonagenarian femoral neck patients varied from 23.3 to 47.6% [6, 16, 30, 33–35]. Although our 1-year mortality rate in the arthroplasty group was lower than those in the previously mentioned studies, it was similar to other studies [11, 36]. Another possible explanation for the differences in mortality rates between this study and previous studies might be related to different demographics, including age, sex distributions, and comorbidities in each study.

Limited studies have demonstrated that five-year mortality in hip fracture patients varies from 55 to 82% [11, 16, 17, 26]. After a retrospective analysis of a total of 149 patients, de Leur et al. found that five-year survival in nonagenarian hip fracture patients was 18% after osteosynthesis [17]. In a large nationwide database study of 11,184 nonagenarian patients undergoing surgery for hip fracture in Taiwan, the mortality rates increased from 29.5% at 1 year to 78.1% at 5 years after surgery [16]. Lin et al. also demonstrated that 10-year mortality was 95.90%, which means that less than 5% of patients survived more than 10 years [16]. The five-year mortality (51.5%) in the arthroplasty group in this study was consistent with the results of Gregory et al. (55%) and Knauf et al. (61.5%) [11, 25] but higher than those in the two previously mentioned studies [16, 17].

The survival time in the arthroplasty group was significantly higher than that in the nonoperative group (53 months versus 22 months, *p* = 0.001). This gap in survival time may be a result of highly selective patient recruitment in that series, resulting in only 33 nonagenarians operated on over 16 years. Large-sample and multicenter studies deserve to be comprehensively evaluated to obtain a clear understanding of long-term survival among these patients.

In our study, we found a higher 5-year survival probability in the arthroplasty group than in the nonoperative treatment group (39.3%,13/33 versus 11.3%,6/53, *p* = 0.002). Manton et al. showed that the five-year survival probability in Japanese individuals aged 90 years ranged from 22.7 to 33.0% [37]. In the Netherlands, the survival rate of people aged 90 years increased from 19.45% in 1990 to 31.4% in 2016 [38]. Compared with the observed survival probability of patients in the current study, the survival rate in the nonoperative group (11.3%) was less than that in the average population, and that in the arthroplasty group (39.3%) was similar to that in the average population. Although the sample size was limited, the results can partially reflect the advantage of surgery for long-term survival among this unique group of patients. This encouraging result supports the rapid increase in healthcare expenditures and infrastructure development and the recent implementation of universal healthcare coverage in China [23].

One unique aspect of this study was that the number of patients who self-selected nonoperative care was higher than that in the arthroplasty group. Although operative treatment for femoral neck fractures is prevalent in developed countries, preoperative discussions with some Chinese patients and family caregivers may be challenging to initiate because of low educational levels or cultural taboos about discussing death [39]. Selection bias regarding patients who underwent arthroplasty may explain some of these disparities; however, there is no doubt that unequal outcomes between China and Western developed countries are multifactorial in origin, and factors such as trust in physicians and medication adherence may play a role [40].

The primary strength of this study was the data collection, low rate of loss to follow-up (< 3%), and duration of follow-up, which was more than 10 years; it was the longest of any published series and exceeded the life expectancy for this age group. This cohort was followed for more than 10 years, which eliminated the bias effect of demographic changes. To the authors’ knowledge, this is the most extensive study to date comparing nonoperative treatment and arthroplasty in patients with femoral neck fracture over 90 years of age.

The interpretation of the findings of our study must comprehensively consider the limitations of our study design. The limitations of our research are inherent to the nature of retrospective reviews performed in a single teaching hospital. Future longitudinal and multicenter studies should be conducted to establish practice guidelines for the treatment of femoral neck fracture in extremely elderly patients. Second, selection bias and other unconscious bias associated with the survival outcome in this study should be considered unique issues. Further research is needed to better understand the effect of these biases on the survival outcome among Chinese or Asian hip fracture patients. Finally, because this study did not include intertrochanteric fractures, our conclusions are not representative of all nonagenarian and centenarian hip fractures.

## Conclusion

Our study suggests that arthroplasty is more likely to improve long-term survival in femoral neck fracture patients over the age of 90 than nonoperative treatment. It can be expected that nearly half of patients will survive more than 5 years after surgery. This subpopulation can be treated similarly to younger elderly patients if initial preoperative assessment suggests comparable health. Although inherent selection bias and unconscious bias existed in this retrospective study, our findings offer valuable information for orthopedic surgeons by providing long-term survival data to enhance critical patient-clinician discussions.

## Data Availability

The datasets used and/or analyzed during the current study are available from the corresponding author on reasonable request.

## Abbreviations

BMI: Body mass index
CCI: Charlson comorbidity index
ASA: American Society of Anesthesiologists
URBMI: The Urban Resident Basic Medical Insurance
UEBMI: The Urban Employee Basic Medical Insurance
NRCMS: The New Rural Cooperative Medical Scheme
